# Architectural constraints on learning and memory function

**DOI:** 10.1186/1471-2202-12-S1-P31

**Published:** 2011-07-18

**Authors:** Ann M Hermundstad, Kevin S Brown, Danielle S Bassett, Jean M Carlson

**Affiliations:** 1 Department of Physics, University of California, Santa Barbara, CA 93117, USA

## 

Recent experimental and computational studies have identified relationships between architecture and functional performance in information processing systems ranging from natural neuronal ensembles [1,2] to artificial neural networks [3,4]. While these systems can vary greatly in their size and complexity, they share certain structural features, such as parallel and layered motifs [5]. Quantifying how these features influence functionality is a first step toward understanding the behavior of both natural and artificial information processing systems. Of particular interest is the impact of structural architecture on the ability of the system to balance stability with flexibility, for example in memory versus learning.

In this study, we use neural networks as model information processing systems to examine tradeoffs in learning and memory processes arising from variations in structural organization. We compare the performance of parallel and layered structures during sequential function approximation, a task that requires networks to produce, retain, and dynamically adapt representations of external information. We measure network performance over a range of learning conditions by statistically analyzing the error in these representations while varying the initial network state, the structure of the external information, and the time allowed for learning. By characterizing local error landscape curvature, we can directly relate the functional performance of the system to its underlying architecture.

Across a range of both parallel and layered system architectures, we find that variations in error landscape curvature give rise to tradeoffs between the ability of these networks to learn new versus retain old information, maximize success versus minimize failure, and produce specific versus generalizable representations of information. In particular, parallel networks generate smooth error landscapes with deep, narrow minima. Therefore, given sufficient time and through the adjustment of a large number of connection weights, parallel networks can find highly specific representations of the external information. Although accurate, however, these representations are difficult to generalize. In contrast, layered networks generate rough error landscapes with a variety of local minima, allowing them to quickly find coarse representations by adjusting a fewer number of weights. Although less accurate, these representations are more easily adaptable.

We have conducted a detailed analysis of network performance over a range of parallel and layered architectures, thereby isolating learning and memory tradeoffs that arise from underlying structural complexity. A thorough understanding of small network systems is crucial for predicting the behavior of larger systems in which statistical studies of performance would not be possible. In particular, these results may provide insight into the behavior of composite systems, such as cortical layers composed of structurally distinct columns [6] or modular divide-and-conquer networks [7], which share features of both parallel and layered architectures. Additionally, the existence of tradeoffs inherent to a range of network structures may help explain the variability of architectural motifs observed in large-scale biological [5] and technical [3] systems. Identifying the structural mechanisms that impact performance has implications for understanding a wide variety of both natural and artificial learning systems.
